# Breastfeeding and later depression and anxiety in mothers in Ireland: a 10-year prospective observational study

**DOI:** 10.1136/bmjopen-2024-097323

**Published:** 2026-01-08

**Authors:** Catherine McNestry, Sharleen L O’Reilly, Patrick J Twomey, Rachel K Crowley, Sophie Callanan, Alice Kasemiire, Alexander Douglass, Anna Delahunt, Fionnuala M McAuliffe

**Affiliations:** 1 UCD Perinatal Research Centre, National Maternity Hospital, Dublin, Ireland; 2 School of Agriculture and Food Science, University College Dublin, Dublin, Ireland; 3 Department of Clinical Pathology, St Vincent’s University Hospital, Dublin, Ireland; 4 School of Medicine and Medical Science, University College Dublin, Dublin, Ireland; 5 Department of Endocrinology, St Vincent’s University Hospital, Dublin, Ireland; 6 Centre for Support and Training in Analysis and Research, University College Dublin, Dublin, Ireland; 7 School of Public Health, Physiotherapy & Sports Science, University College Dublin, Dublin, Ireland

**Keywords:** Physiology, MENTAL HEALTH, Maternal medicine, Postpartum Women

## Abstract

**Objectives:**

Although breastfeeding is associated with lower postnatal depression and anxiety, limited research exists regarding long-term maternal mental health outcomes. This study examined the association between breastfeeding and depression and anxiety in women of later reproductive age (mid 30s to menopause).

**Design:**

This was a 10-year prospective longitudinal cohort study. Self-reported questionnaires were used to collect lifetime breastfeeding behaviour at 10 years, and health history including depression, anxiety and medication use was collected at each study timepoint.

**Setting:**

A tertiary level maternity hospital in Dublin, Ireland.

**Participants:**

168 parous women from the ROLO Longitudinal Cohort with lifetime breastfeeding behaviour and health history data available at 10 years were included (22% of total cohort). Women currently pregnant or breastfeeding at 10-year follow-up were excluded.

**Results:**

Mean (SD) age at study end was 42.4 (3.8) years. 72.6% (n=122) of women reported ever breastfeeding. Median lifetime exclusive breastfeeding was 5.5 weeks (IQR 35.8, range 0–190). 37.5% of women (n=63) breastfed for ≥12 months over their lifetime. 13.1% (n=22) reported depression or anxiety at 10 years, and 20.8% (n=35) reported depression or anxiety over the whole study period. Ever breastfeeding was associated with less depression and anxiety at 10 years (OR 0.34, 95% CI 0.12 to 0.94, p=0.04). Ever breastfeeding, longer exclusive breastfeeding and lifetime breastfeeding ≥12 months were associated with lower depression and anxiety over the whole study period (ever breastfeeding OR 0.4, p=0.03; exclusive breastfeeding OR 0.98/week, p=0.03; lifetime breastfeeding ≥12 months OR 0.38, p=0.04).

**Conclusion:**

There may be a protective association between breastfeeding and self-reported depression and anxiety. Further studies are required to confirm the findings.

**Trial registration number:**

ISRCTN54392969.

**STRENGTHS AND LIMITATIONS OF THIS STUDY**

- This is a 10-year observational longitudinal cohort study of 168 healthy multiparous mothers.
- We used patient-reported outcomes and controlled for baseline between-group differences.
- Findings are limited by the small numbers, observational data and relatively homogenous study population.
- Causality cannot be inferred by observational data and further studies are required to confirm the findings.

## Introduction

 Breastfeeding is associated with multiple physical health benefits for both mother and infant. Infant benefits include reduced infections and sudden infant death syndrome in the first year of life, and lower risk of obesity and chronic disease in later life compared with formula fed infants.^1^ Evidence also suggests that breastfeeding has a positive association with infant mental health and cognitive function, including improved infant attachment security, reduced behavioural disorders and improved academic attainment later in childhood.^2^,^5^ Maternal physical health benefits include lower risk of later cardiovascular disease, breast and ovarian cancer and type 2 diabetes.^1 6^

Regarding mothers’ mental health, it is accepted that there is a relationship between breastfeeding and mood disorders, notably postnatal depression. This relationship is bidirectional; pregnancy and postpartum depression predict earlier breastfeeding cessation, but breastfeeding may mediate the association between depression in pregnancy and postnatal depression.^7^ Two recent systematic review and meta-analyses have confirmed an association between breastfeeding and reduced postnatal depression, with a more pronounced effect found in mothers who breastfeed exclusively.^8 9^ Postnatal anxiety is also associated with poorer breastfeeding outcomes, although the evidence is low quality.^10^ Moreover, anxiety and depression are closely related—often co-existing or pre-existing one another—and have overlapping symptoms, so similar outcomes and effects are not unexpected.^11 12^

The physiology behind the association between breastfeeding and postpartum anxiety and depression may lie in the effects of the lactation hormones. Oxytocin decreases maternal anxiety and promotes mother and infant bonding.^13^ Lower oxytocin levels in pregnancy can impair the emotional adaptation to motherhood and are associated with increased depressive symptoms postpartum.^9 14^ Lactation hormones may promote normal maternal hypothalamic-pituitary-adrenal axis functioning.^15^

Limited evidence exists on whether there is a relationship between breastfeeding and maternal depression and/or anxiety outside of the postpartum period.^16^ It is known that postnatal depression is associated with poorer maternal mental health in the longer term,^17^ hence if breastfeeding is associated with a lower risk of postnatal depression, there may also be an association with lower incidence of depression and anxiety in later life. This study examines the association between breastfeeding and depression and anxiety in a 10-year longitudinal cohort of parous women of later reproductive age, which we define as mid 30s to menopause.

## Methods

### The ROLO study

The ROLO Longitudinal Cohort originated with the ROLO study, a randomised controlled trial of a low-glycaemic index diet to prevent recurrence of macrosomia. Women with a history of delivering a >4 kg infant were recruited early in their second pregnancy. A prior history of gestational diabetes, any other significant medical history or use of any medications in pregnancy were exclusion criteria. Full details of the original study and outcomes have been previously published.^18^ Mother and child pairs were invited back at 3 and 6 months, 2, 5 and 10 years postpartum for ongoing study of cardiometabolic, nutritional and general health outcomes. Demographics and body mass index were collected by trained researchers at the first study visit (12–14 weeks of pregnancy).^18^

### Exposure: breastfeeding behaviour

An Infant Feeding questionnaire was sent to all participants attending a 10-year study visit to gather complete lifetime maternal breastfeeding behaviour. This was a modified version of a validated infant feeding questionnaire.^19^ Researcher interest led to the introduction of the Infant Feeding questionnaire at a late stage of follow-up. Efforts were made by email, post and telephone to offer all participants still in the study the opportunity to complete it. Four data points were examined: ever breastfeeding (breastfed or expressed milk for 1 day or longer over the lifetime), total lifetime weeks of exclusive breastfeeding, total lifetime weeks of any breastfeeding, and lifetime breastfeeding of ≥12 months versus <12 months. The 12-month period was selected because it has been linked to other long-term maternal health benefits associated with lifetime breastfeeding exposure.^6^ Women currently pregnant or breastfeeding at the time of the 10-year study visit were excluded from the analysis.

### Outcome: participant-reported anxiety and depression

At each follow-up time point, participants completed a detailed health history questionnaire. This included a specific yes/no question about whether the person had received a diagnosis of depression, a free text answer for ‘other’ conditions and a free text answer for ‘current medications’. Participants positively reporting a diagnosis of depression and/or anxiety, or reporting taking antidepressant medication, were deemed positive for depression and anxiety. Two outcomes were examined: depression and anxiety reported at 10 years, and depression and anxiety reported at any follow-up.

### Covariates and demographics

Baseline and 10-year covariate data were used for this analysis. Potential covariates were selected based on known risk factors for mood disorders and author consensus.^20 21^ Baseline differences between groups were controlled for in the statistical model,^22^ as well as alcohol intake at 10 years. Ten-year data were used to control for alcohol intake, as the baseline data were from pregnancy, when many women do not drink alcohol or significantly reduce its intake.

The Pobal HP (Haase-Pratschke) social deprivation index was calculated for participants using baseline demographics.^23^ Parity, smoking status and last menstrual period were collected via questionnaire at each follow-up time point. Baseline and last menstrual period and contraception history at 10 years were used to determine menopausal status. Menopause was defined as no menstrual period for at least 12 months in the absence of use of hormonal contraception.

Dietary intake was assessed using the updated 2007 SLÁN 150-item food frequency questionnaire.^24^ To analyse daily quantities and nutrient composition of each item within the questionnaire, standard food portion sizes were assigned using the food standards portions sizes book.^25^ An updated version of McCance and Widdowson’s composition of foods integrated dataset was used for nutrient analysis.^26^ From this, alcohol intake was calculated in grams per day.

Physical activity was measured at baseline using the 1998 SLÁN questionnaire and used to calculate estimated metabolic equivalent of tasks (METs) expended per week.^27^ One MET is the amount of energy expended when sitting quietly.^28^

Participants completed the World Health Organization-5 (WHO-5) index at baseline and at each follow-up. The WHO-5 index is a validated five-item questionnaire used to assess well-being and quality of life and is presented in this work as a percentage score.^29^

### Statistical analysis

The Kolmogorov-Smirnov test and histograms were used to assess data distribution for normality. Independent t tests were used to compare the central tendency of groups at baseline and at 10 years, and χ^2^ tests were used to test categories at the same time points. Fisher’s exact test was used where the assumptions of the χ^2^ test were violated. Mann-Whitney U test was used instead of t-tests for non-normally distributed data. Forced-entry logistic regression models were developed to examine further the impact of breastfeeding behaviour on mental health outcomes. Baseline differences between groups were controlled for in model 1, and model 2 additionally controlled for alcohol intake at 10 years. Linear regression was used to calculate Variance Inflation Factors for variables in model 2 of each logistic regression to test for multicollinearity.

Statistical analysis was carried out using SPSS (Statistical Package for the Social Sciences) for Mac version 17 (IBM, San Francisco, CA, USA).

### Patient and public involvement

The ROLO Longitudinal Cohort Study was an early adopter of Patient and Public Involvement via the ROLO Family Advisory and Young Person’s Advisory Committees, which have been important in helping to direct our research.^30 31^

### Consent to Participate

Did participants give informed consent to participate in the study before taking part?

## Results

Of the original 759 women included in the ROLO randomised controlled trial, 437 (57%) returned at 10 years: 205 of these women filled in the maternal infant feeding behaviour questionnaire (27%); and 168 women had complete lifetime breastfeeding and health history data available at 10 years postpartum and made up this study cohort (22%).

At 10 years, the mean (SD) age of participants was 42.4 (3.8) years (range 31–50). All participants were pregnant with their second child at recruitment, and by 10 years 56.5% (n=95) had no further children. Parity ranged from 2 to 5 at 10 years (para 3, 30.4%, n=51; para 4, 11.3%, n=19; para 5, 1.8%, n=3); 95.2% of women were White Irish (n=160); 63.1% had completed third level education (n=94) at study entry; and 98.7% of women remained premenopausal at 10 years (n=156).

Regarding breastfeeding, 72.6% of women reported ever breastfeeding (n=122) at 10 years, with a median lifetime exclusive breastfeeding duration of 5.5 weeks (IQR 35.8, range 0–190) and a median lifetime any breastfeeding duration of 30.5 weeks (IQR 84.0, range 0–488); 37.5% of women reported cumulative lifetime breastfeeding of ≥12 months (n=63).

Regarding depression and anxiety, 13.1% of women reported depression/anxiety at 10 years (n=22), with 20.8% reporting depression or anxiety at any time point (n=35).

Participant demographics at baseline and at 10 years are presented in Tables1  2, respectively. Table 2 includes study outcomes of participant-reported depression and anxiety at 10 years and at any time over the study period, as well as a summary of participants’ breastfeeding behaviour.

**Table 1:** Characteristics at study entry (early pregnancy)

	n	Mean (SD)	Range
Age (years)	168	32.4 (3.71)	22–40
Ethnicity (White Irish)^*^	168	160 (95.2%)	–
Completed third level education^*^	149	94 (63.1%)	–
Social deprivation (Pobal HP) index	168	5.36 (9.32)	−23.8 to 24.7
METs per week^†^	146	365 (404)	0–1662
Smoking history^*^	166		
Never		85 (51.2%)	–
Ex		76 (45.8%)	–
Current		5 (3.0%)	–
Well-being (WHO-5) % score	149	56.4 (14.0)	8–100
BMI^†^	166	25.48 (5.1)	18.2–45.3
BMI category^*^	166		
Underweight		1 (0.6%)	–
Normal		73 (44.0%)	–
Overweight		63 (38.0%)	–
Obese		29 (17.5%)	–
RCT group (intervention)^*^	168	86 (51.2%)	–

Data are presented as mean (SD) for normally distributed data,and median (IQR) for non-normally distributed data. Categorical data are presented as number (%)

* n (%).

† Median (IQR).

BMI, body mass index; HP, Haase-Pratschke; METs, metabolic equivalents; RCT group, group assigned in the ROLO randomised controlled trial.

**Table 2:** Characteristics at 10 years and lifetime breastfeeding behaviour

	n	Mean (SD)	Range
Age (years)	168	42.4 (3.8)	31–50
Parity^*^	168		
2		95 (56.5%)	–
3		51 (30.4%)	–
4		19 (11.3%)	–
5		3 (1.8%)	–
Premenopausal^*^	158	156 (98.7%)	–
Well-being (WHO-5) % score^†^	167	64 (20)	16–100
Alcohol intake (g per day)†	166	4.1 (10.5)	0–72.5
METs per week^†^	146	365 (404)	0–1662
Smoking^*^	168		
Never		85 (50.6%)	–
Ex		70 (41.7%)	–
Current		13 (7.7%)	–
Depression/anxiety at 10 years^*^	168	22 (13.1%)	–
Depression/anxiety at any follow-up*	168	35 (20.8%)	–
Ever breastfed^*^	168	122 (72.6%)	–
Exclusive breastfeeding (weeks)^†^	166	5.5 (35.8)	0–190
Any breastfeeding (weeks)^†^	168	30.5 (84.0)	0–488
Lifetime breastfeeding ≥12 months^*^	168	63 (37.5%)	–

Data are presented as mean (SD) for normally distributed data, and median (IQR) for non-normally distributed data. Categorical data are presented as number (%)

* n (%).

† Median (IQR).

METs, metabolic equivalents.

Univariate analysis was performed to compare groups at baseline and at 10 years. Participant-reported depression and anxiety at 10 years was associated with younger age, lower physical activity levels and lower well-being score at baseline (mean (SD) age 30.9 (4.1) years vs 32.7 (3.6) years, p=0.03; median (IQR) METs per week 278 (254) vs 405 (439), p=0.04; median (IQR) WHO-5 well-being score 52% (22%) vs 57.5% (14.1%), p=0.02). A difference in age and well-being score persisted at 10 years, but a difference in physical activity levels did not (median (IQR) METs per week at 10 years 266 (451] vs 365 (404), p=0.16). Women reporting depression and anxiety at any time point differed only by age at baseline from women not reporting any depression and anxiety (mean (SD) age 31.0 (4.0) years vs 32.8 (3.6) years, respectively, p=0.01).

Women reporting depression and anxiety at 10 years were more likely to be smokers at 10 years on univariate analysis (current smoker 22.7% (n=5/22) vs 5.5% (n=8/146), p=0.02). There was no difference in parity, menopausal status, body mass index, alcohol intake or physical activity levels. At 10 years, women reporting depression and anxiety at any follow-up had similar findings when compared with women not reporting depression and anxiety (current smoker 20.0% (n=7/35) vs 4.5% (n=6/133), p=0.006; mean (SD) WHO-5 score 57% (16%) vs 64% (16%), p=0.03).

Women reporting depression and anxiety at 10 years were less likely to have breastfed (ever breastfed 50% (n=11/22) vs 76% (n=111/146), p=0.02), and had shorter durations of any or exclusive breastfeeding over their lifetime (median (IQR) exclusive breastfeeding 0 (6) weeks vs 9 (41) weeks, p=0.002; median (IQR) any breastfeeding 0 (15.5) weeks vs 37 (86.5) weeks, p=0.004). This was also the case for women reporting depression and anxiety at any follow-up time (ever breastfeeding 54.3% (n=19/35) vs 77.4% (n=103/133), p=0.01; median (IQR) exclusive breastfeeding 0 (12) weeks vs 12 (42) weeks, p=0.003; median (IQR) any breastfeeding 0 (32) weeks vs 38 (91) weeks, p=0.001; lifetime breastfeeding ≥12 months 20.0% (n=7/35) vs 42.1% (n=56/133), p=0.03).

Logistic regression analysis results showing the association of ever breastfeeding and breastfeeding duration with depression and anxiety outcomes are presented in online supplemental tables 1 and 2. Following correction for baseline between-group differences, the association remained between ever breastfeeding and depression and anxiety at 10 years (OR 0.34, p=0.04), and ever breastfeeding (OR 0.4, p=0.03), exclusive breastfeeding (OR 0.98, p=0.03) and lifetime breastfeeding >12 months (OR 0.383, p=0.04) and depression and anxiety over the study period (tables3  4).

**Table 3:** Adjusted associations between depression and anxiety at 10 years and lifetime breastfeeding behaviour

	B	SE	P value	OR	95% CI
Ever breastfed	−1.08	0.519	**0.04**	0.34	0.12 to 0.94
Exclusive breastfeeding (weeks)	−0.017	0.013	0.2	0.98	0.96 to 1.01
Any breastfeeding (weeks)	−0.003	0.004	0.5	1.0	0.99 to 1.01
Lifetime breastfeeding ≥12 months	−0.664	0.617	0.28	0.52	0.15 to 1.72

Forced-entry binary logistic regression used for analysis.

Adjusted for baseline age, WHO-5 wellbeing score, physical activity and for alcohol intake at 10 years. P<0.05

SE, standard error of B.

**Table 4:** Adjusted associations between depression and anxiety at any follow up and lifetime breastfeeding behaviour

	B	SE	P value	OR	95% CI
Ever breastfed	−0.915	0.419	**0.03**	0.40	0.18 to 0.91
Exclusive breastfeeding (weeks)	−0.021	0.009	**0.03**	0.98	0.96 to 0.997
Any breastfeeding (weeks)	−0.007	0.004	0.06	0.99	0.99 to 1.00
Lifetime breastfeeding ≥12 months	−0.959	0.468	**0.04**	0.383	0.15 to 0.96

Forced-entry binary logistic regression used for analysis.

Adjusted for baseline age and alcohol intake at 10 years. P<0.05.

SE, standard error of B.

Variance inflation factors for covariates examined ranged from 1.001 to 1.15. These are presented in online supplemental table 3.

## Discussion

We found that ever breastfeeding, ≥12 months lifetime breastfeeding and lifetime exclusive breastfeeding were all associated with a lower likelihood of self-reported depression and anxiety over the course of the 10-year study period. Each week of lifetime exclusive breastfeeding was associated with a 2% lower likelihood of reporting depression and anxiety. This association persisted after controlling for baseline differences between groups and alcohol intake. Ever breastfeeding was associated with lower risk of reporting depression and anxiety at 10 years timepoint, but differences were not found in the other reported outcomes after controlling for confounders. As far as we are aware, this is the first time such a relationship has been described in women of later reproductive age.

The association between breastfeeding and postnatal depression is now well established, but there is a dearth of research examining the effects of breastfeeding on mental health in later life.^16^ Park and Choi described an association between history of breastfeeding and depression in postmenopausal women.^32^ They found depression risk decreased with increasing breastfeeding duration and number of breastfed infants.^32^ Hahn-Holbrook *et al* also describe lower depressive symptoms in women who breastfed more frequently up to 24 months postpartum in their prospective study.^33^ Our findings add to the evidence that breastfeeding is associated with positive maternal mental health outcomes in the longer term. The metabolic syndrome has also been linked to early cessation of breastfeeding and also to mood disorders,^34^ so a predisposition to obesity and related chronic diseases may explain this association. We suggest there also may be a protective effect of successful breastfeeding on postpartum depression and anxiety, which in turn lowers the risk of maternal depression and anxiety in the longer term. The likelihood is that the association is multifactorial, as many socioeconomic and cultural factors influence both breastfeeding and mental health in addition to the impact of health history. Additionally, women with a prior history of depression and anxiety are at risk of lower breastfeeding success, compounding the association but in the reverse direction.^35^

Our research group has previously described positive outcomes for infants and children from breastfeeding.^34 36^ Notably, in the ROLO Longitudinal Cohort offspring, children who breastfed for longer had less emotional eating at 5 years old.^34^ Meta-analysis has also shown reduced odds of later overweight and obesity in breastfed children by 15–27%.^37^ In our cohort, maternal well-being during pregnancy at recruitment was not found to influence breastfeeding outcomes; we believe this reflects the fact that this cohort was healthy and well at baseline and strengthens the later association found.^38^ Baseline between-group differences were controlled for, as particularly physical activity levels and WHO well-being score as a screening tool for depression could influence the outcomes examined.^39 40^

The relationship between breastfeeding and more positive mental health outcomes should inform individualised breastfeeding support for women at risk of postnatal depression, such as women with a history of previous pregnancy loss.^41^ Because pre-existing depression is associated with reduced breastfeeding duration, additional support and resources may be required by these women to help them reach their breastfeeding goals. There is evidence that breastfeeding promotion and behavioural interventions reduce postnatal depression, even when the intervention focus is solely on supporting breastfeeding.^42 43^ We know that improving breastfeeding rates and duration can improve lifetime health outcomes, reducing population level disease burden and resulting in significant healthcare savings.^44^,^46^ The possibility that breastfeeding could further reduce the huge burden of depression on individuals, families, healthcare systems and economies only adds to the argument for policymakers to improve breastfeeding support.^47^ Breastfeeding needs further promotion and exploring antenatal breastfeeding efficacy, including societal support, and instigating effective interventions to support women to breastfeed holds promise to promote life-long health.^48^,^50^

One of the strengths of this study is the rich 10-year dataset, allowing us to examine multiple potential confounders and to control for baseline differences between groups. Another strength is our purposefully recruited healthy population with no medical background history. Examining a healthy cohort at baseline reduces the potential confounding effect of pre-existing physical and mental health problems.

The study is limited by the relatively small number of participants, which may have resulted in some of the analyses being underpowered. The observational nature of the data means that causality cannot be inferred from the results, although a randomised controlled trial of breastfeeding versus formula feeding would be unethical given the known benefits of breastfeeding for women and children. The study is also limited by infrequent and irregular follow-up time points across a 10-year period postpartum. While the use of antidepressant drugs was deemed positive for depression and anxiety, it should be acknowledged that the reason for taking this medication was not recorded and could have been prescribed for other conditions such as pain. The study also did not capture engagement with alternative treatment options for depression and anxiety such as counselling. Some results may therefore be attributed to unmeasured or residual confounding factors, which should be explored in greater depth in future work.

Another limitation worth noting is the patient-reported outcomes which are open to recall bias and under-reporting, particularly in the case of depression and anxiety.^51^ However, evidence suggests that women can accurately recall their breastfeeding history for up to 20 years postpartum.^52 53^ Use of a composite outcome variable is intended to negate some of the effects of under-reporting and recall bias. Self-selection bias is also a possible limitation, as women with depression and anxiety, or those who did not or could not breastfeed, may have declined to complete the 10-year questionnaires. Finally, as this cohort is relatively homogenous in terms of ethnicity and educational attainment, the findings may not be applicable to women who had only one child, women who experienced significant medical problems before or during pregnancy, women who have not had a macrosomic infant, and other groups not represented by the study population.

Future studies should include larger prospective studies to confirm our findings and to define the underlying pathways that result in the association described. Validated outcome measures could be used to capture the data in future studies. Studies in women with pre-existing anxiety and depression, and in first-time mothers, would also be beneficial to see if the favourable association with mental health outcomes persists. Similarly, studies examining interventions designed to improve breastfeeding outcomes in women with depression should consider including longer-term outcomes in their findings.

## Conclusion

Ever breastfeeding may be associated with reduced rates of depression and anxiety in multiparous women of later reproductive age at 10 years postpartum. Longer exclusive breastfeeding, ever breastfeeding and cumulative lifetime breastfeeding of 12 months or greater may be associated with reduced diagnosis of maternal depression and anxiety over the following 10 years. This association, previously unreported in this age group, only strengthens the recommendation that breastfeeding is the optimal infant feeding method, for both mother and baby. Further studies are required to explore potential benefits for pregnant women with a history of anxiety and depression.

## Supplementary material

10.1136/bmjopen-2024-097323 online supplemental file 1

## Data Availability

Data are available on reasonable request from the corresponding author.
