# Supplementary material for: Breastfeeding and later depression and anxiety in mothers in Ireland: a 10-year prospective observational study
Source: BMJ Open. 2026 Jan 8;16(1):e097323. doi: 10.1136/bmjopen-2024-097323 (PMC13059848; doi:10.1136/bmjopen-2024-097323)
Supplement: online supplemental file 1 [file bmjopen-16-1-s001.docx]

Supplementary Table 1. Associations between depression and anxiety at 10 years and lifetime breastfeeding behaviour

|  | Unadjusted | | | | | Model 1 | | | | | Model 2 | | | | |
| --- | --- | --- | --- | --- | --- | --- | --- | --- | --- | --- | --- | --- | --- | --- | --- |
|  | B | S.E. | p | OR | 95% CI | B | S.E. | p | OR | 95% CI | B | S.E. | p | OR | 95% CI |
| Ever breastfed | -1.154 | 0.468 | **0.01** | 0.32 | 0.13, 0.79 | -1.092 | 0.519 | **0.04** | 0.34 | 0.12, 0.93 | -1.08 | 0.519 | **0.04** | 0.34 | 0.12, 0.94 |
| Exclusive breastfeeding (weeks) | -0.027 | 0.014 | 0.05 | 0.97 | 0.95, 1.0 | -0.17 | 0.013 | 0.21 | 0.98 | 0.96, 1.01 | -0.017 | 0.013 | 0.2 | 0.98 | 0.96, 1.01 |
| Any breastfeeding (weeks) | -0.007 | 0.005 | 0.15 | 0.99 | 0.99, 1.0 | -0.003 | 0.004 | 0.54 | 0.99 | 0.99, 1.01 | -0.003 | 0.004 | 0.5 | 1.0 | 0.99, 1.01 |
| Lifetime breastfeeding ≥ 12 months | -1.116 | 0.578 | 0.05 | 0.33 | 0.11, 1.02 | -0.625 | 0.613 | 0.31 | 0.54 | 0.16, 1.78 | -0.664 | 0.617 | 0.28 | 0.52 | 0.15, 1.72 |
| Forced-entry binary logistic regression used for analysis. SE = Standard error of B. OR = Odds ratio. 95% CI = 95% confidence interval of odds ratio. Model 1 adjusted for baseline age, WHO-5 wellbeing score and physical activity. Model 2 additionally adjusted for alcohol intake at 10 years. **p <0.05** | | | | | | | | | | | | | | | |

Supplementary Table 2. Associations between depression and anxiety at any follow up and lifetime breastfeeding behaviour

|  | Unadjusted | |  |  |  | Model 1 | |  |  |  | Model 2 | | | | |
| --- | --- | --- | --- | --- | --- | --- | --- | --- | --- | --- | --- | --- | --- | --- | --- |
|  | B | SE | p | OR | 95% CI | B | SE | p | OR | 95% CI | B | S.E. | p | OR | 95% CI |
| Ever breastfed | -1.062 | 0.398 | **0.008** | 0.35 | 0.16, 0.75 | -0.86 | 0.414 | **0.04** | 0.42 | 0.19, 0.95 | -0.915 | 0.419 | **0.03** | 0.40 | 0.18, 0.91 |
| Exclusive breastfeeding (weeks) | -0.025 | 0.01 | **0.02** | 0.98 | 0.96, 0.99 | -0.022 | 0.01 | **0.02** | 0.98 | 0.96, 0.99 | -0.021 | 0.009 | **0.03** | 0.98 | 0.96,0.997 |
| Any breastfeeding (weeks) | -0.008 | 0.004 | **0.04** | 0.99 | 0.98, 0.99 | -0.007 | 0.004 | 0.06 | 0.99 | 0.98, 1.00 | -0.007 | 0.004 | 0.06 | 0.99 | 0.99, 1.00 |
| Lifetime breastfeeding ≥ 12 months | -1.068 | 0.458 | **0.02** | 0.34 | 0.14, 0.84 | -0.969 | 0.464 | **0.04** | 0.38 | 0.15, 0.94 | -0.959 | 0.468 | **0.04** | 0.383 | 0.15,0.96 |
| Forced-entry binary logistic regression used for analysis. SE = Standard error of B. OR = Odds ratio. 95% CI = 95% confidence interval of odds ratio. Model 1 adjusted for baseline age. Model 2 additionally adjusted for alcohol intake at 10 years. **p <0.05** | | | | | | | | | | | | | | | |

| **Supplementary Table 3. Variance Inflation Factors for Logistic Regression Model Variables** | | | | | |  | | | |  |
| --- | --- | --- | --- | --- | --- | --- | --- | --- | --- | --- |
|  |  | |  | | |  | | | |  |
| Depression/anxiety at 10 years | | |  | | |  | | | |  |
| Ever breastfed | | Baseline age | | | Baseline WHO-5 Wellbeing Score | | | Baseline physical activity | | 10-year Alcohol intake |
| 1.082 | | 1.115 | | | 1.05 | | | 1.082 | | 1.0165 |
| Exclusive breastfeeding (weeks) | | Baseline age | | | Baseline WHO-5 Wellbeing Score | | | Baseline physical activity | | 10-year Alcohol intake |
| 1.028 | | 1.036 | | | 1.059 | | | 1.102 | | 1.021 |
| Any breastfeeding (weeks) | | Baseline age | | | Baseline WHO-5 Wellbeing Score | | | Baseline physical activity | | 10-year Alcohol intake |
| 1.066 | | 1.036 | | | 1.058 | | | 1.15 | | 1.018 |
| Lifetime breastfeeding ≥ 12 months | | Baseline age | | | Baseline WHO-5 Wellbeing Score | | | Baseline physical activity | | 10-year Alcohol intake |
| 1.056 | | 1.048 | | | 1.06 | | | 1.106 | | 1.024 |
|  |  | |  | | |  | | | |  |
| Depression/anxiety at any follow up | | |  | | |  | | | |  |
| Ever breastfed | | | | Baseline age | | | 10-year alcohol intake | |  |  |
| 1.082 | | | | 1.078 | | | 1.003 | |  |  |
| Exclusive breastfeeding (weeks) | | | | Baseline age | | | 10-year alcohol intake | |  |  |
| 1.008 | | | | 1.002 | | | 1.007 | |  |  |
| Any breastfeeding (weeks) | | | | Baseline age | | | 10-year alcohol intake | |  |  |
| 1.004 | | | | 1.001 | | | 1.004 | |  |  |
| Lifetime breastfeeding ≥ 12 months | | | | Baseline age | | | 10-year alcohol intake | |  |  |
| 1.029 | | | | 1.02 | | | 1.009 | |  |  |
