# Supplementary material for: Breastfeeding and later depression and anxiety in mothers in Ireland: a 10-year prospective observational study
Source: BMJ Open. 2026 Jan 8;16(1):e097323. doi: 10.1136/bmjopen-2024-097323 (PMC13059848; doi:10.1136/bmjopen-2024-097323)
Supplement: Supplementary file 2 [file bmjopen-2024-097323.reviewer_comments.pdf]

PEER REVIEW HISTORY

BMJ Open publishes all reviews undertaken for accepted manuscripts. Reviewers are asked to complete a checklist review form and are provided with free text boxes to elaborate on their assessment. These free text comments are reproduced below.

ARTICLE DETAILS

Title (Provisional)

Breastfeeding and later depression and anxiety in Irish mothers: a 10-year prospective observational study

Authors

McNestry, Catherine; O'Reilly, Sharleen; Twomey, Patrick J; Crowley, Rachel; Callanan, Sophie; Kasemiire, Alice; Douglass, Alexander; Delahunt, Anna; McAuliffe, Fionnuala

---

VERSION 1 - REVIEW

**Reviewer**	**1**
**Name**	**Toledo, Christine**
**Affiliation**	**Florida Atlantic University**
**Date**	**04-Jan-2025**
**COI**	**None**

Abstract- In the conclusion (you state) the association of breastfeeding Improved mental health outcomes. However, all women were healthy at baseline with no prior health history so this would imply a protective relationship, not an improvement.

Background- It would be helpful to the readers to define what is considered “later life” depression/anxiety and studies that are related to “later life” mental health

Methods- While I understand the parent study was cited, including basic information such as the inclusion and exclusion criteria would be beneficial. From my understanding, the study only included women who were not primiparous but had already had a child before with no self-reported prior medical history? It was not as clear. For the sample size reduction- it was not clear why only 168 out of 432 women completed lifetime breastfeeding. Did they just not want to fill it out or were not asked?

A power analysis to understand if sample size was sufficient for analysis power would have been appropriate and reporting the effect size.

Covariates-It is not clear how they selected the specific covariates and demographic variables. While it was defined, selection is not clear if it was based on prior literature.

Analysis- usually for such models SEM would be appropriate, I am guessing due to the smaller sample size this analysis was not able to be used?

Results- Line 228 you meant “ever” breastfeeding instead of “even” breastfeeding?

Discussion- You state in Line 248 that this is the first time such a relationship is described in this age group, but you did not clearly define this age group, and I was able to find studies looking at this relationship in women in their 40s as women are giving birth later in life.

Lines 250 to 268 discussing oxytocin and the HPA pathway seems out of place as you did not examine oxytocin as a variable and did not test this theory in your study. The information seems out of place. Perhaps you can succinctly describe this as the theoretical framework to the relationship in the background. However, this is not a finding of your study, nor does it add support to this theory.

Lines 267-268 seem repetitive.

You cited only one study in the discussion but there are other studies using longitudinal data looking at the same relationship that you could have discussed.

Lines 273-274 talk about oxytocin again....

Lines 293-295 speak about breastfeeding recommendations with a history of depression, but your study included only healthy women. I would speak more towards the protective theory to prevent mental health issues.

Strengths/Limitations- Line 314 states that findings are relevant to the general population. In Ireland? The demographics show that the sample was predominantly White Irish. Also, from my understanding, it did not include first time mothers (a population that has higher postpartum anxiety and depression than multiparas). Additionally, since the outcome was self-reported diagnosis and medications, the outcome may have been under-reported since many could have had symptoms of anxiety and depression during the 10 years and never sought treatment.

**Reviewer**	**2**
**Name**	**Morriss, Richard**
**Affiliation**	**University of Nottingham, Psychiatry**
**Date**	**28-May-2025**
**COI**	**None**

The current manuscript has examined the effects of breast feeding on maternal depression and anxiety over 10 years. Since there are few longer-term cohort studies that have reported data on this topic then the current manuscript provides some useful data. However, there are some important limitations of this data that are not sufficiently explored or acknowledged. These limitations need to be discussed since there is little that can be done now to rectify them. I will return to these.

Another important issue to address is that the background of the abstract and the paper claim that the study examined the association between breastfeeding and depression and

anxiety in later reproductive age women. However, the design of the study was not based on age but on having a previous large baby so all the mothers had a previous child (so they would be a bit older than a sample that included first-time mothers as well). The age range was 22-40 years, therefore including some younger mothers. The authors have selectively focussed on one of the characteristics of the sample rather than a design feature of the study. It should be reported as an observation rather than a hypothesis, which implies that the study was prospectively designed to explore older mothers.

There are a number of important limitations that pose a threat to the internal validity of the study and a number that limit its generalisability. Only 22% of the original sample are reported here, with a large proportion excluded because they could not provide a breast feeding history. This is an important selection bias based on the ability to recall information. Depression and anxiety affect the ability to recall periods of their life so it is possible that there were women with a history of depression who had breast fed who were excluded because they cannot recall the information on breast feeding to the exacting requirements of the research team (recollection of all periods of breast feeding in exact number of weeks). It is important to know what women were told about the study in relation to the effects of breast feeding. If there was any suggestion that breast feeding was good for maternal and child health, including maternal mental health, then this might bias who was willing to take part in the study and what they reported about both breast feeding. and their depression or anxiety.

Recall bias might be a substantial problem as well. Breast feeding data was only collected retrospectively at 10 years with an expected accuracy of one week. Women may struggle to remember in this much detail what they did in relation to breast feeding, especially as these women were recruited when they were having 2nd, 3rd or 4th child so their breast feeding history would have extended much further than 10 years.

Thirdly, there is also the problem of ascertainment bias. There is a large discrepancy between the report women gave of a diagnosis of depression or anxiety and treatment with antidepressants at 10 years than the report at each follow up point when these reports are aggregated. There was no checking of these reports with primary care and specialist mental health service records. There was no exploration of these discrepancies in the reporting of depression and anxiety or any explanation for them. The 10 year data would be more likely to be subject to recall bias so more likely to underestimate the true outcome in comparison to the data collected at each follow up. Furthermore the latter data before 10 years would not have been linked to reporting of breast feeding at 10 year. There is also the problem that antidepressants are not just used for depression and anxiety but have other widespread uses such as pain and to aid sleep. Furthermore depression and anxiety is often treated psychologically and a lot of that treatment in Ireland would be obtained privately or through self-treatment using digital or bibliotherapy methods. Another issue is that a lot of depression and anxiety is never diagnosed or treated by health care staff or not disclosed even if they have been because of stigma. I would have expected in this situation for there to

be more testing of the data on depression and anxiety in sensitivity analyses to examine if the association with breast feeding remains if only a diagnosis of depression or anxiety disorder was made since this seems to me to be a more specific outcome than one based on antidepressant use.

Fourthly there are threats to the internal validity of the findings due to the statistical approaches. Well-being and physical activity are highly confounded by depression and the former also by low self-esteem, a risk factor for depression. Women with depression or low self-esteem will report low well-being and tend to be underactive so these are not independent variables. There should be tests for multicollinearity. Similarly depressed women will also tend to have a higher BMI, come from more deprived areas, smoke and drink more so the model should consider all of these variables as potential confounders. Testing univariate associations is not necessarily a robust way of selecting which of these variables should be in the logistic regression model and which should not ; there might be interactions between these variables or even latent traits linking several of these that would not appear in a univariate analysis since all of these variables have bidirectional effects with both depression and breast feeding. Breast feeding is associated with education, less deprivation and healthier lifestyles. Other confounders not reported that might be important include whether the mother is a single parent or not at the time of index birth and at 10 years, the number of children they have, their physical health, and if they are employed or not.

Since depression and anxiety diagnosis and medication were measured at each time point, another statistical approach to have considered might have been breast feeding in the first 6 months and in previous pregnancies and time to first diagnosis of depression and anxiety. Given the bidirectional relationship between depression and anxiety and breast feeding, a lot of depression or antidepressant use might discourage breast feeding. Given that depression and anxiety are so often recurrent or only partially remits, then time to the first episode of depression and anxiety partly controls

this confounding by history of depression and anxiety. If breast feeding is protective, then it should take longer for these women to develop depression or anxiety.

There are important issues to acknowledge in terms of external validity or generalisability. First the results do not generalise to women who have never been pregnant before or never had a heavy baby before. They do not generalise to women who would never have agreed to take part in the trial such as those with low health literacy or who are not fluent in English. The majority of women had higher education (readers may not be familiar with the term third education) and the sample has little ethnic diversity being 95% white Irish. All of these issues of generalisability should be acknowledged.

VERSION 1 - AUTHOR RESPONSE

Reviewer 1

Thank you for your detailed comments on our manuscript. We hope you find the edits satisfactory. Please find a point-by-point breakdown below.

Abstract- In the conclusion (you state) the association of breastfeeding Improved mental health outcomes. However, all women were healthy at baseline with no prior health history so this would imply a protective relationship, not an improvement.

Thank you for this comment, we have edited the conclusion as follows:

"There was a protective association between breastfeeding and mental health outcomes in later reproductive age women. This is a novel finding in this age group, with further studies required to confirm the association and its directionality."

Background- It would be helpful to the readers to define what is considered "later life" depression/anxiety and studies that are related to "later life" mental health

Thank you for this comment, we have edited this sentence as follows:
"Limited evidence exists on whether there is a relationship between breastfeeding, and maternal depression and/or anxiety outside of the postpartum period."

We have also clarified what we define as later reproductive age later in the Introduction as follows:
"This study examines the association between breastfeeding and depression and anxiety in a 10-year longitudinal cohort of parous women of later reproductive age, which we define as mid 30s to menopause."

Methods- While I understand the parent study was cited, including basic information such as the inclusion and exclusion criteria would be beneficial. From my understanding, the study only included women who were not primiparous but had already had a child before with no self-reported prior medical history? It was not as clear.

Thank you for this comment. Yes, your understanding is correct. We believe we have adequately discussed the inclusion and exclusion criteria of the original study in lines 98-102:

"The ROLO Longitudinal Cohort originated with the ROLO Study, a randomised controlled trial of a low-glycaemic index (GI) diet to prevent recurrence of macrosomia. Women with a history of delivering a >4kg infant were recruited early in their second pregnancy. A prior history of gestational diabetes, any other significant medical history or use of any medications in pregnancy were exclusion criteria."

We have edited lines 104-105 as follows, and hope this makes the origin of this cohort more clear:

"Mother and child pairs were invited back at 3 and 6 months, 2, 5 and 10 years postpartum for ongoing study of cardiometabolic, nutritional and general health outcomes."

For the sample size reduction- it was not clear why only 168 out of 432 women completed lifetime breastfeeding. Did they just not want to fill it out or were not asked?

Thank you for this comment. We have added to the Sample Size section for further detail:

"Of the original 759 women included in the ROLO randomised controlled trial, 432 (57%) returned at 10 years. 205 of these women filled in the maternal infant feeding behaviour questionnaire (27%). Researcher interest led to the introduction of this. questionnaire at a late stage of follow up. Efforts were made by email, post and telephone to offer all participants still in the study the opportunity to complete it. 168 women had complete lifetime breastfeeding and health history data available at 10 years postpartum and made up the study cohort (22%)."

A power analysis to understand if sample size was sufficient for analysis power would have been appropriate and reporting the effect size.

Thank you for this comment. A power analysis was not performed for the secondary analysis of the ROLO study described in our manuscript. The recent literature suggests that post-hoc power analysis is theoretically flawed, where sample size cannot be changed and data is already collected (Zhang et al., General Psychiatry, 2019 https://doi.org/10.1136/gpsych-2019-100069*; Althouse, The journal of surgical research, 2021.* https://doi.org/10.1016/j.jss.2019.10.049*). As retrospective power calculations are not thought to add any benefit to the research (Althouse, 2021), we used an exploratory research design instead.*

Covariates-It is not clear how they selected the specific covariates and demographic variables. While it was defined, selection is not clear if it was based on prior literature.

Thank you for this comment. We have expanded on the selection of covariates and added the appropriate citations to the references as follows:

"Baseline and 10-year covariate data was used for this analysis. Potential covariates were selected based on known risk factors for mood disorders and author consensus. Baseline differences between-groups were controlled for in the statistical model , as well as alcohol intake at 10 years. 10-year data was used to control for alcohol intake, as the baseline data was from pregnancy, when many women do not drink alcohol or significantly reduce it."

Analysis- usually for such models SEM would be appropriate, I am guessing due to the smaller sample size this analysis was not able to be used?

Thank you for this comment. As the outcomes (depression/anxiety at 10 years and depression/anxiety at any follow-up) are binary variables, logistic regression was used instead of multiple linear regression.

Results- Line 228 you meant "ever" breastfeeding instead of "even" breastfeeding?

Thank you – yes, this has been edited accordingly.

Discussion- You state in Line 248 that this is the first time such a relationship is described in this age group, but you did not clearly define this age group, and I was able to find studies looking at this relationship in women in their 40s as women are giving birth later in life.

Thank you for this comment. We would be interested to know the studies you found. A systematic review by Bugaeva et al. in 2023 identified only 3 studies specifically looking at maternal mental health outside of the postpartum period. One being the study we cite by Park and Choi , another looking at depression up to 24 months postpartum (Hahn-Holbrook et al., 2014) where the mean maternal age was 29 years. The third looked at hospital admissions for postpartum mental disorders up to 12 months postpartum, which we would argue is different to later depression and anxiety outside of postpartum. We have added a definition of "later reproductive age" to the manuscript as above.

Lines 250 to 268 discussing oxytocin and the HPA pathway seems out of place as you did not examine oxytocin as a variable and did not test this theory in your study. The information seems out of place. Perhaps you can succinctly describe this as the theoretical framework to the relationship in the background. However, this is not a finding of your study, nor does it add support to this theory. Lines 267-268 seem repetitive.

Thank you for this suggestion. We have edited this paragraph for brevity, as below, and moved it to the Introduction. Lines 267-268 have been deleted.

"The physiology behind the association between breastfeeding and postpartum. anxiety and depression may lie in the effects of the lactation hormones. Oxytocin decreases maternal anxiety and promotes mother and infant bonding. Lower oxytocin levels in pregnancy can impair the emotional adaptation to motherhood, and is associated with increased depressive symptoms postpartum. Lactation hormones may promote normal maternal hypothalamic-pituitary-adrenal axis functioning."

You cited only one study in the discussion but there are other studies using longitudinal data looking at the same relationship that you could have discussed.

Thank you for this comment. We have added the following reference to this section:

"Hahn-Holbroook et al. also describe lower depressive symptoms in women who breastfed more frequently up to 24 months postpartum in their prospective study."

Lines 273-274 talk about oxytocin again….

Thank you for this comment, these lines have been deleted.

Lines 293-295 speak about breastfeeding recommendations with a history of depression, but your study included only healthy women. I would speak more towards the protective theory to prevent mental health issues.

Thank you for this comment. We have removed this mention and edited the relevant lines as follows:

"The relationship between breastfeeding and more positive mental health outcomes should inform individualized breastfeeding support for women at-risk of postnatal depression such as women with a history of previous pregnancy loss [35]."

Strengths/Limitations- Line 314 states that findings are relevant to the general population. In Ireland? The demographics show that the sample was predominantly White Irish. Also, from my understanding, it did not include first time mothers (a population that has higher postpartum anxiety and depression than multiparas).

Thank you for this comment, we have edited to remove the reference to the 'general population' as follows:

“Another strength is our purposefully recruited healthy population with no medical background history.”

We have acknowledged that our cohort is not representative of all women further below:

“as this cohort is relatively homogenous in terms of ethnicity and educational attainment, the findings may not be applicable to women who had only one child, women who experienced significant medical problems before or during pregnancy, women who have not had a macrosomic infant or other groups not represented by the study population”

Additionally, we have edited the recommendations for future studies:

“Studies in women with pre-existing anxiety and depression and in first-time mothers would also be beneficial to see if the favourable association with mental health outcomes persists.”

Additionally, since the outcome was self-reported diagnosis and medications, the outcome may have been under-reported since many could have had symptoms of anxiety and depression during the 10 years and never sought treatment.

Thank you for this comment, we have edited to include this point in the Limitations:

“Another limitation worth noting are the patient-reported outcomes, which are open to recall bias and under-reporting, particularly in the case of depression and anxiety.”

Reviewer: 2

Thank you for your detailed commentary on our manuscript. We hope you find the edits satisfactory. Please find a point-by-point response below,

Comments to the Author:
The current manuscript has examined the effects of breast feeding on maternal depression and anxiety over 10 years. Since there are few longer-term cohort studies that have reported data on this topic then the current manuscript provides some useful data. However, there are some important limitations of this data that are not sufficiently explored or acknowledged. These limitations need to be discussed since there is little that can be done now to rectify them. I will return to these.

Another important issue to address is that the background of the abstract and the paper claim that the study examined the association between breastfeeding and depression and anxiety in later reproductive age women. However, the design of the study was not based on age but on having a previous large baby so all the mothers had a previous child (so they would be a bit older than a sample that included first-time mothers as well). The age range was 22-40 years, therefore including some younger mothers. The authors have selectively focussed on one of the characteristics of the sample rather than a design feature of the study. It should be reported as an observation rather than a hypothesis, which implies that the study was prospectively designed to explore older mothers.

Thank you for this comment. We have edited the text in the Introduction as follows to remove the reference to hypothesis:

"It is known that postnatal depression is associated with poorer maternal mental health in the longer term, hence if breastfeeding is associated with a lower risk of postnatal depression, there may also be an association with lower incidence of depression and anxiety in later life. This study examines the association between breastfeeding and depression and anxiety in a 10-year longitudinal cohort of parous women of later reproductive age, which we define as mid 30s to menopause."

We have edited the Abstract and Introduction to clarify the reference to later reproductive age women. We intended this to refer to the participants' age at study end rather than entry.

“This study examined the association between breastfeeding and depression and anxiety in women of later reproductive age (mid 30s to menopause).”

“This study examines the association between breastfeeding and depression and anxiety in a 10-year longitudinal cohort of parous women of later reproductive age, which we define as mid 30s to menopause.”

We have also edited the Methods (The ROLO Study) section for further clarity:

“Mother and child pairs were invited back at 3 and 6 months, 2, 5 and 10 years postpartum for ongoing study of cardiometabolic, nutritional and general health outcomes.”

There are a number of important limitations that pose a threat to the internal validity of the study and a number that limit its generalisability. Only 22% of the original sample are reported here, with a large proportion excluded because they could not provide a breast feeding history. This is an important selection bias based on the ability to recall information. Depression and anxiety affect the ability to recall periods of their life so it is possible that there were women with a history of depression who had breast fed who were excluded because they cannot recall the information on breast feeding to the exacting requirements of the research team (recollection of all periods of breast feeding in exact number of weeks).

Thank you for this comment. Rates of depression in our cohort align with the lifetime incidence of depression (20%), although this figure itself is prone to underestimation (Kelly et al., Irish Journal of Medical Science, 2019). We have edited the Limitations to strengthen this point as follows:

“Another limitation worth noting is the patient-reported outcomes which are open to recall bias and under-reporting, particularly in the case of depression and anxiety. Self-selection bias is also a possible limitation, as women with depression and

anxiety, or those who did not or could not breastfeed may have declined to complete the 10-year questionnaires."

It is important to know what women were told about the study in relation to the effects of breast feeding. If there was any suggestion that breast feeding was good for maternal and child health, including maternal mental health, then this might bias who was willing to take part in the study and what they reported about both breast feeding. and their depression or anxiety.

Thank you for this comment. As this is a subanalysis of a larger prospective observational study, specific information was not provided to participants linking breastfeeding and maternal mental health. As detailed in the Methods, these were collected in separate questionnaires. Although every effort was made to not exclude mothers who formula fed (by choice or not), this is certainly a possibility. We used a validated questionnaire with a neutral title purposefully for this reason. However, the reported rates of ever breastfeeding we found in the subset who responded to the Infant Feeding questionnaire (72.6%) is not hugely higher than the rates of breastfeeding initiation in these mothers (63.3-64.6%; as reported by Horan et al., 2014) and may be mostly accounted for by those mothers who breastfed their first baby, for however short or long a time, and subsequently did not initiate breastfeeding for their second child.

Recall bias might be a substantial problem as well. Breast feeding data was only collected retrospectively at 10 years with an expected accuracy of one week. Women may struggle to remember in this much detail what they did in relation to breast feeding, especially as these women were recruited when they were having 2nd, 3rd or 4th child so their breast feeding history would have extended much further than 10 years.

Thank you for this comment. For accuracy, we had excluded women who were pregnant or breastfeeding at the time of the 10-year study visit, and have added this to the Methods (Sample Size) section:

"Women currently pregnant or breastfeeding at the time of 10-year study visit were excluded."

As above, we have listed recall bias as a potential limitation in the study already. Interestingly, evidence suggests that women can recall their breastfeeding history with a high degree of accuracy up to 10-20 years later (Natland et al., BMC Medical Research Methodology, 2012, https://doi.org/10.1186/1471-2288-12-179*, van Zyl et al., Journal of Human Nutrition and Dietetics, 2016,* https://doi.org/10.1111/jhn.12384*). We had previous data available regarding the second infant of our cohort only (not the first or subsequent children), and this was cross-checked with the 10-year responses.*

Thirdly, there is also the problem of ascertainment bias. There is a large discrepancy between the report women gave of a diagnosis of depression or anxiety and treatment with antidepressants at 10 years than the report at each follow up point when these reports are aggregated. There was no checking of these reports with primary care and specialist mental health service records. There was no exploration of these discrepancies in the reporting of depression and anxiety or any explanation for them. The 10 year data would be more likely to be subject to recall bias so more likely to underestimate the true outcome in comparison to

the data collected at each follow up. Furthermore the latter data before 10 years would not have been linked to reporting of breast feeding at 10 year. There is also the problem that antidepressants are not just used for depression and anxiety but have other widespread uses such as pain and to aid sleep. Furthermore depression and anxiety is often treated psychologically and a lot of that treatment in Ireland would be obtained privately or through self-treatment using digital or bibliotherapy methods. Another issue is that a lot of depression and anxiety is never diagnosed or treated by health care staff or not disclosed even if they have been because of stigma. I would have expected in this situation for there to be more testing of the data on depression and anxiety in sensitivity analyses to examine if the association with breast feeding remains if only a diagnosis of depression or anxiety disorder was made since this seems to me to be a more specific outcome than one based on antidepressant use.

Thank you for this comment. We do not have consent from participants to validate the reported information with other healthcare practitioners, so this is not possible. We have acknowledged the possibility of recall bias and under-reporting in the study limitations. The authors agreed on use of the composite variable including use of antidepressant medication

to try to counteract the effects of recall bias and non-disclosure, however we acknowledge your important point. We have added this clause to the Limitations:

"Use of a composite outcome variable is intended to negate some of the effects of under-reporting and recall bias."

Fourthly there are threats to the internal validity of the findings due to the statistical approaches. Well-being and physical activity are highly confounded by depression and the former also by low self-esteem, a risk factor for depression. Women with depression or low self-esteem will report low well-being and tend to be underactive so these are not independent variables. There should be tests for multicollinearity.

Thank you for this comment. We have addressed multicollinearity by adding VIF scores in Supplementary table 1 and the following additions to Methods and Results:

"Linear regression was used to calculate Variance Inflation Factors for variables in model 2 of each logistic regression to test for multicollinearity."
"Variance Inflation Factors for covariates examined ranged from 1.001 to 1.15. These are presented in Supplementary Table 1."

Similarly depressed women will also tend to have a higher BMI, come from more deprived areas, smoke and drink more so the model should consider all of these variables as potential confounders. Testing univariate associations is not necessarily a robust way of selecting which of these variables should be in the logistic regression model and which should not ; there might be interactions between these variables or even latent traits linking several of these that would not appear in a univariate analysis since all of these variables have bidirectional effects with both depression and breast feeding. Breast feeding is associated with education, less deprivation and healthier lifestyles. Other confounders not reported that might be important include whether the mother is a single parent or not at the time of index birth and at 10 years, the number of children they have, their physical health, and if they are employed or not.

Thank you for this comment. Certainly, any number of covariates may be examined. We used Social Deprivation Index rather than employment history, and have reported number of

children (as Parity) in Table 2. As discussed in the Methods, all participants were healthy at baseline. We selected those variables that differed at baseline due to concerns regarding non-convergence of the models if too many variables were controlled for, as our study number is small. A similar approach has been mentioned by Trutchel et al. (2017):

"the choice of which variables should be included in the model can be based on the degree of significance in the difference between the treatment and control groups in the baseline analysis" (Section 3.2.3)

Since depression and anxiety diagnosis and medication were measured at each time point, another statistical approach to have considered might have been breast feeding in the first 6 months and in previous pregnancies and time to first diagnosis of depression and anxiety. Given the bidirectional relationship between depression and anxiety and breast feeding, a lot of depression or antidepressant use might discourage breast feeding. Given that depression and anxiety are so often recurrent or only partially remits, then time to the first episode of depression and anxiety partly controls this confounding by history of depression and anxiety. If breast feeding is protective, then it should take longer for these women to develop depression or anxiety.

Thank you for this comment. Our data does not allow for us to match timing of diagnosis to timing of birth of each infant, but we will certainly consider this approach for future studies.

There are important issues to acknowledge in terms of external validity or generalisability. First the results do not generalise to women who have never been pregnant before or never had a heavy baby before. They do not generalise to women who would never have agreed to take part in the trial such as those with low health literacy or who are not fluent in English. The majority of women had higher education (readers may not be familiar with the term third education) and the sample has little ethnic diversity being 95% white Irish. All of these issues of generalisability should be acknowledged.

Thank you for this comment. We have edited the Limitations section as follows:

“Finally, as this cohort is relatively homogenous in terms of ethnicity and educational attainment, the findings may not be applicable to women who had only one child, women who experienced significant medical problems before or during pregnancy, women who have not had a macrosomic infant or other groups not represented by the study population.”

VERSION 2 - REVIEW

**Reviewer**	**2**
**Name**	**Morriss, Richard**
**Affiliation**	**University of Nottingham, Psychiatry**
**Date**	**14-Aug-2025**
**COI**	

This is one of the few studies that have examined breast feeding and longitudinal maternal mental health so publication is worth consideration. However, I have serious reservations about the methodology and whether the study might be biased or misleading. I don't think there is much the authors can do except improve some of the wording.

The authors say that this is an exploratory study in their response to providing a retrospective sample size. There are also problems with the outcome measurement as these are not validated outcomes for the measure depression and anxiety such as the PHQ-9, GAD-7, Edinburgh Postnatal Depression Scale. Self-report at unequally spaced follow up points and the use of antidepressant drugs (which are used for other purposes such as pain) but not other types of depression treatment like counselling is of unknown validity in relation to depression and anxiety. The ascertainment of breast feeding occurred sometime later into the study so open to recall bias with recall being a problem when there is depression and anxiety and asking for such detail retrospectively when around 44% women had 3 or more previous pregnancies. There was a low response rate with only 27% of the sample filling out the maternal infant feeding behaviour questionnaire and only 22%of the original cohort completing the questionnaire. None of this information is conveyed in the abstract. Participants may have been open to selection bias since dropout from follow up was more likely in people who had depression or anxiety and could have happened before completion of the maternal infant feeding behaviour questionnaire. There are numerous potential measured and unmeasured confounders that might explain any association. As explained before the relationship between breast feeding and depression is bidirectional but the methodology and analysis assumes a unidirectional relationship.

However, the paper is not written as if it is an exploratory study and the claims are far too bold.

For instance the title should read "breast feeding may be associated with reduced self-reported depression and anxiety", not is associated and not depression and anxiety without the qualification of self-report.

The abstract conclusion should say "there might be a protective association between breastfeeding and self-reported depression and anxiety which requires further research", not there was an association between breastfeeding and mental health outcomes in later reproductive age women. As stated before the cohort was not defined specifically by age but by being multiparous, which is important because there are known differences between first time pregnancy and multiparous women on depression and anxiety. The conclusion of the main paper should state that ever breastfeeding may be associated with reduced rates of depression and anxiety in multiparous older women at 10 years postpartum.

VERSION 2 - AUTHOR RESPONSE

Reviewer: 2
Prof. Richard Morriss, University of Nottingham
Comments to the Author:
This is one of the few studies that have examined breast feeding and longitudinal maternal mental health so publication is worth consideration. However, I have serious reservations about the methodology and whether the study might be biased or misleading. I don't think there is much the authors can do except improve some of the wording.

The authors say that this is an exploratory study in their response to providing a retrospective sample size. There are also problems with the outcome measurement as these are not validated outcomes for the measure depression and anxiety such as the PHQ-9, GAD-7, Edinburgh Postnatal Depression Scale.

Thank you for this comment. We have acknowledged this point by adding the following sentence to the Discussion:

"Validated outcome measures could be used to capture the data in future studies."

Self-report at unequally spaced follow up points and the use of antidepressant drugs (which are used for other purposes such as pain) but not other types of depression treatment like counselling is of unknown validity in relation to depression and anxiety.

Thank you for this comment. We have further added to the Limitations as follows:

"The study is limited by infrequent and irregular follow-up time points across a 10-year period postpartum. While the use of antidepressant drugs was deemed positive

for depression and anxiety, it should be acknowledged that the reason for taking this medication was not recorded and could have been prescribed for other conditions such as pain. The study also did not capture engagement with alternative treatment options for depression and anxiety such as counselling.”

The ascertainment of breast feeding occurred sometime later into the study so open to recall bias with recall being a problem when there is depression and anxiety and asking for such detail retrospectively when around 44% women had 3 or more previous pregnancies.

Thank you for this comment. We have further addressed this point with the addition of the following comment within the Limitations:

“However, evidence suggests that women can accurately recall their breastfeeding history for up to 20 years postpartum [52, 53].”

There was a low response rate with only 27% of the sample filling out the maternal infant feeding behaviour questionnaire and only 22%of the original cohort completing the questionnaire. None of this information is conveyed in the abstract.

Thank you for this comment. We hope our previous edits to the study limitations have acknowledged many of the above points sufficiently:

“The study is limited by the relatively small number of participants, which may have resulted in some of the analyses being underpowered. The observational nature of the data means that causality cannot be inferred from the results, although a randomized controlled trial of breastfeeding versus formula feeding would be unethical given known benefits of breastfeeding for women and children.
Another limitation worth noting is the patient-reported outcomes which are open to recall bias and under-reporting, particularly in the case of depression and anxiety [51]. However, evidence suggests that women can accurately recall their breastfeeding history for up to 20 years postpartum [52, 53]. Use of a composite outcome variable is intended to negate some of the effects of under-reporting and recall bias. Self-selection bias is also a possible limitation, as women with depression and anxiety, or those who did not or could not breastfeed may have declined to complete the 10-year questionnaires. Finally, as this cohort is relatively homogenous in terms of ethnicity and educational attainment, the findings may not be applicable to women who had only one child, women who experienced significant

medical problems before or during pregnancy, women who have not had a macrosomic infant and other groups not represented by the study population."

Additionally, we have further edited the abstract to include more information about the study methods as follows:

"Design 10-year prospective longitudinal cohort study. Self-reported questionnaires were used to collect lifetime breastfeeding behaviour at 10 years, and health history including depression, anxiety and medication use were collected at each study timepoint.

Setting A tertiary level maternity hospital in Dublin, Ireland

Participants 168 parous women from the ROLO Longitudinal Cohort with lifetime breastfeeding behaviour and health history data available at 10 years were included (27% of total cohort). Women currently pregnant or breastfeeding at 10-year follow up were excluded."

Participants may have been open to selection bias since dropout from follow up was more likely in people who had depression or anxiety and could have happened before completion of the maternal infant feeding behaviour questionnaire. There are numerous potential measured and unmeasured confounders that might explain any association.

Thank you for this comment. We have further added to the Limitations to address this point:

"Some results may therefore be attributed to unmeasured or residual confounding factors, which should be explored in greater depth in future work."

As explained before the relationship between breast feeding and depression is bidirectional but the methodology and analysis assumes a unidirectional relationship.

Thank you for this comment. We have added the following phrase to the Discussion:

*"*Additionally, women with a prior history of depression and anxiety are at risk of lower breastfeeding success, compounding the association but in the reverse direction*"*

to further acknowledge the bidirectional nature of the relationship between breastfeeding and mental health.

However, the paper is not written as if it is an exploratory study and the claims are far too bold.
For instance the title should read "breast feeding may be associated with reduced self-reported depression and anxiety", not is associated and not depression and anxiety without the qualification of self-report.

Thank you for this comment. We have altered the title to read:

Breastfeeding and later depression and anxiety in Irish mothers: a 10-year prospective observational study

The abstract conclusion should say "there might be a protective association between breastfeeding and self-reported depression and anxiety which requires further research", not there was an association between breastfeeding and mental health outcomes in later reproductive age women.

Thank you for this comment. We have edited the abstract conclusion as suggested:

"There may be a protective association between breastfeeding and self-reported depression and anxiety. Further studies are required to confirm the findings."

As stated before the cohort was not defined specifically by age but by being multiparous, which is important because there are known differences between first time pregnancy and multiparous women on depression and anxiety. The conclusion of the main paper should state that ever breastfeeding may be associated with reduced rates of depression and anxiety in multiparous older women at 10 years postpartum.

Thank you for this comment. We have edited the main paper conclusion as suggested:

"Ever breastfeeding may be associated with reduced rates of depression and anxiety in multiparous older women at 10 years postpartum. Longer exclusive breastfeeding, ever breastfeeding, and cumulative lifetime breastfeeding of 12 months or greater may be associated with reduced diagnosis of maternal depression and anxiety over the following 10 years."

Thank you again for consideration of our edited manuscript. I look forward to hearing from you soon.

VERSION 3 - REVIEW

**Reviewer**	**2**
**Name**	**Morriss, Richard**
**Affiliation**	**University of Nottingham, Psychiatry**
**Date**	**25-Sep-2025**
**COI**	

The authors have addressed my comments from my previous two reviews.
